# Paraoxonase: Its antiatherogenic role in chronic renal failure

**DOI:** 10.4103/0971-4065.62088

**Published:** 2010

**Authors:** M. Prakash, N. M. Phani, R. Kavya, M. Supriya

**Affiliations:** Department of Biochemistry, Kasturba Medical College, Manipal - 576 104, India

**Keywords:** Atherosclerosis, chronic renal failure, hemodialysis, high density lipoprotein, low density lipoprotein oxidation, paraoxonase

## Abstract

Paraoxonase (PON) is an aryldialkylphosphatase, which reversibly binds and hydrolyzes organophosphates. The PON family has three members (PON1, PON2 and PON3); they share structural properties and enzymatic activities. PON1 is shown to reside over high density lipoprotein (HDL) and has both antioxidant and antiatherogenic functions. Function of PON2 and PON3 are speculative and still under research. Several methodologies were developed over the years to determine the activity and mass of PON1, of which spectrophotometer-based methods using certain chemicals as substrate predominate. Several studies have shown decreased levels of PON1 in chronic renal failure (CRF) patients, particularly those on hemodialysis. The role of PON1 in development of cardiovascular disease has drawn considerable attention in recent years. Several authors have shown decreased levels of HDL and PON1 activity in CRF patients on hemodialysis and reported this to be a risk factor in the development of CVD. Enhancement or maintenance of the PON1 activity may prevent development of CVDs and its consequences in patients on hemodialysis.

## Introduction

Paraoxonase (PON) (EC 3.1.8.1, aryldialkylphosphatase) is a protein of 354 amino acids with a molecular mass of 43 kDa.[1,2] There are three known genotypic forms of PON which are coded by the set of genes *PON1, PON2* and *PON3*, respectively, and are located on long arm of chromosome 7.[3–5] PON1 is synthesized in liver and transported in plasma by binding to high density lipoprotein (HDL); PON2 is ubiquitously expressed intracellular protein;[6] and PON3 is similar to PON1 in activity but differs from it in substrate specificity. [7] The exact function of the different family members is not clear, although the conservation among the individual family members across species suggests a strong evolutionary pressure to preserve these functional differences.[5] Studies performed during last ten years indicate that PON has multiple functions. PON protects low density lipoprotein (LDL) from oxidative modification by reactive oxygen species (ROS), and thus, significantly contributes to the atheroprotective effect of HDL.[8–10]

The enzyme also hydrolyzes phospholipid hydroperoxides and cholesterol hydroperoxides (esterase activity) and reduces lipid peroxides to respective hydroxides as well as degrades peroxides (peroxidase activity).[11] PON1 by binding to HDL protects it from peroxidation, and hence, improves reverse cholesterol transport.[12] PON1 also protects plasma membrane from free radical injury.[13] Furthermore, PON1 degrades bioactive phospholipids, such as platelet activating factor, thereby preventing intravascular coagulation.[14] Recent studies have indicated that PON1 possesses lactonase activity and is involved in the metabolism of statins, spironolactone, and glucocorticoid lactones.[ 15] It hydrolyses homocysteine thiolactone and prevents homocysteinemia, a process involved in atherogenesis.[16] The product of PON2 has not yet been identified in biological tissue, but the PON3 gene product has recently been identified as a lactonase located on rabbit HDL.[17]

The preferential association of PON1 with HDL is mediated in part by its signal peptide.[5] Binding of PON1 to HDL helps in transport across the plasma membrane of HDL or phospholipid expressing cells.[5] Major part of this enzyme in serum is associated with HDL particles.[18] Although, apolipoprotein A-I (Apo A-I) is not necessary for PON1 association with HDL, but its activity is stabilized in the presence of the Apo A-I. Only in the absence of both lecithin cholesterol acyltransferase (LCAT) and apolipoprotein E (Apo E) PON1 is associated with non-HDL lipoproteins.[7] In these settings PON1 is found to be associated with very low density lipoprotein (VLDL) and chylomicrons.[11,19] No PON1 is bound to LDL particles,[20] although PON1 is indirectly involved in protection of LDL from oxidation and hence in the prevention of atherosclerosis.[21]

PON2 is absent in plasma but is expressed in many tissues.[6] PON3 is found to be associated with HDL particles.[7,17] Both PON2 and PON3 posses antioxidant properties and lactonase activity, but unlike PON1 they lack paraoxon or phenyl acetate hydrolyzing activity.[22] However, Draganov *et al*. have observed PONs exhibiting overlapping and distinct substrate specificities.[23] Authors reported that only PON1 shown organophosphatase activity (acting on substrates like paraoxon and diazoxon), while arylesterase activity (acting on substrates like phenyl acetate) and overlapping lactonase activity were shown by all the three PONs. However, it was reported that PON2 hydrolyzed *N*-acyl-homoserine lactones, while bulky drug substrates such as lovastatin and spironolactone are hydrolyzed only by PON3.[23]

There is wide inter-individual variation in the capacity of PON1 to hydrolyze organophosphates and other organic esters.[5] In contrast, organophosphates are suicidal inhibitors of other organic esterase, such as pseudo cholinesterase in serum and acetyl cholinesterase at synapses and the neuromuscular junctions, because they bind irreversibly to them.[13] Thus, PON1 is the main means of protection of the nervous system against the neurotoxicity of organophosphates entering the circulation. It was first discovered in this context and its name reflects its ability to hydrolyze paraoxon, a metabolite of the insecticide parathion.[13] PON1 levels are influenced by a variety of environmental factors, including statins and cytokines.[5] Statins increase PON1 activity by up-regulating hepatic PON1 expression.[5] Expression of cellular PON2 was also found to be up-regulated by statins. Nutritional antioxidants, such as polyphenols, increase PON1 mRNA expression and activity by an aryl hydrocarbon receptor-dependent mechanism.[6]

## Paraoxonase Assay

Various methods have been developed to determine PON1 activity; the earliest were the spectrophotometer based methods using different chemicals as substrate for the enzyme. Schiavon *et al*.[24] and Paragh *et al*.[25] have determined PON1 activity using paraoxon (O,O-diethyl-O-*p*-nitrophenyl phosphate) as the substrate. Hasselwander *et al*. measured the PON1 activity using phenylacetate as substrate.[26] The PON phenotype distribution was determined by the dual-substrate method,[27] which calculates the ratio of salt-stimulated PON1 activity at Ph 10.5 and arylesterase activity.[28] PON1 requires calcium for activity and is inactivated in the presence of ethylene diamine tetracetic acid (EDTA). Because of this, studies to date have used serum or heparinized plasma for both activity and mass assays of PON1.[29]

Whole serum and EDTA plasma were analyzed by SDS-electrophoresis and western blot using anti PON1 monoclonal antibody 4C10. Because PON1 has one disulfide and one free cysteine residue, the samples were reduced with dithiothreitol before electrophoresis. [29] Western blot identified a major PON1 band with a molecular mass of 45 kDa and two minor bands of 40 and 35 kDa in both serum and EDTA plasma.[29] This established that PON1 is inactive, but structurally intact in EDTA plasma and suggested that a mass assay could be developed based on SDS-electrophoresis and western blot.[29] Kujiraoka *et al*. developed a sensitive sandwich enzyme-linked immunosorbent assay (ELISA), using two monoclonal antibodies against PON1, to measure serum PON1 concentration.[30] In recent times PON1 paraoxonase activity in serum is determined by a highly sensitive fluorometric assay (excitation/emission maxima 360/450 nm), for the organophosphate activity of PON-1, based on the hydrolysis of a fluorogenic organophosphate analog (Molecular Probes, Eugene, OR). This method has increased specificity and sensitivity and has advantages over other substrates, such as phenylacetate. The average intra-assay CV for PON1 activity using this method is 1.9%.[31]

## Role of paraoxanase in atherosclerosis

PON1 expression is partly controlled by its molecular variation at gene locus.[32] Two polymorphic sites have been described in the coding region: a leucine (L) to methionine (M) transition at position 55 (L55M) and a glutamine (Q) to arginine (R) transition at position 192 (Q192R). The L55M polymorphism affects the enzyme concentration, whereas the Q192R polymorphism affects the catalytic efficiency, but not the concentration.[33,34] Four polymorphisms in the promoter sequence of the PON1 gene (107C/T, 162A/G, 824G/A, 907G/C) also contribute to the variability in protein expression.[35] PON Q and PON R may act on different substrates generated during LDL oxidation and may possess different sensitivities to the action of peroxides formed during LDL oxidation.[36] These differences may contribute to the divergence in the possible antiatherosclerotic roles of the PON allozymes.[36] It has been suggested that the active site in PON1 that protects LDL differs from the active sites for its paraoxone and aryl esterase activities.[36]

Sorenson *et al*. have demonstrated that PON is a lipid-dependent enzyme; in fact, the conformation of PON within the hydrophobic environment of HDL is crucial for its activity. Phospholipids, especially those with long fatty acid chains, stabilize PON enzyme and are required for binding of PON1 at the lipoprotein surface.[37] The PON1 breakdown lipid peroxides before they could accumulate on LDL[8,9,38] and protects against coronary artery disease there by preventing oxidation of LDL.[39] Oxidized LDL is known to possess atherogenic and pro-inflammatory properties.[40] On the other hand HDL protects against CVD by means of reverse cholesterol transport.[40] Even though physiological role of PON1 *in vivo* remains to be clarified, the inhibition of both LDL and HDL oxidation may contribute to protection against CVD.[35]

In addition to genetic influences, PON1 concentration and activity could be modified by lifestyle determinants such as smoking,[41,42] vitamin C and E consumption,[43] and alcohol intake.[44] Therefore, studying PON1 levels and activity in conjunction with variation at the gene level gives a more complete view of the role of PON1 in the development of atherosclerosis.[37] There is also considerable interest in the potential pharmacological effects on PON1 activity. Although there is conflict in findings about the role of lipid lowering drugs on activity of PON1, few studies report increase in PON1 activity by fibric acid derivatives[45,46] and statin.[47] Though, polyphenols in mice have shown to increase serum PON1 activity but such findings are not consistent in human beings.[48]

## Role of PON1 in preventing atherosclerosis in chronic renal failure patients on hemodialysis

CVD is the major cause of morbidity and mortality in patients with chronic renal failure (CRF) and accounts for up to 50% of all deaths.[49] CRF is frequently associated with disturbances in lipoprotein transport, alterations in lipoprotein concentration, and abnormalities in lipid and apoprotein composition of lipoproteins.[50–53] The activities of key enzymes in the lipoprotein metabolism (lipoprotein lipase, hepatic lipase, lecithin-cholesterol acyltransferase) may be diminished.[54–56] This increased susceptibility in these patients is partly explained by increased LDL oxidation and enhance atherogenesis.[28] The pathogenesis of CVD in CRF is multifactorial, including several risk factors.[57,58] But the exact cause for increased susceptibility of CRF patients for atherogenesis is still under investigation.

Several studies have shown decreased activity of PON1 in CRF patients, particularly on maintenance hemodialysis.[59] The decrease in PON1 activity, hence the reduction in its antioxidant and antiatherogenic properties could be an essential factor for premature vascular aging.[59] The decrease in PON1 activity could be the result of lower HDL concentrations in CRF patients, given that HDL is the main serum carrier of PON1. The studies have shown that HDL concentration and phenotypic distribution of may not be the only determining factors.[25] Other possible explanations for the decrease in PON1 activity in CRF patients may be unfavorable uremic environment due to the retention of uremic toxins and or “middle molecules” including advanced glycation endproducts (AGE), free adducts and peptides could play a mechanistic role in decreasing PON1 activity.[60,61] If these molecules are proved to be causal then it will open new treatment option in preventing development of CVDs by designing drugs against these molecules. On the other hand the possibility of an endogenous circulating inhibitors of PON in blood of CRF patients was dismissed by few authors.[24,28,61]

There are few studies on PON1 activity in Indian scenario, Prakash *et al*.[62] have shown significant decreased PON1 activity in CRF patients on conservative management. Decrease was more significant in CRF patients on hemodialysis therapy. Authors have also reported significant positive correlation PON1 with HDL and other antioxidants like protein thiols, and negative correlation with LDL and lipidhydroperoxises. Other authors also reported similar decrease in PON1 activity in CRF patients on conservative management, and they reported a good correlation between serum creatinine and lipid hydroperoxides whereas a negative relationship was seen between PON1 and protein thiols.[63] Similarly, Krishnaswamy *et al*. have reported significant decrease in PON1 activity in CRF patients on hemodialysis and peritoneal dialysis, however, they found normal PON1 levels in renal transplant patients. They have also reported significant increase in antibodies to oxidized LDL in hemodialysis group compared to peritoneal dialysis and transplant subjects.[64]

Schiavon *et al*.[65] found that the serum PON1 activity was significantly reduced in uremic patients. They also report that altered HDL subfraction is likely to be the main cause of the decreased PON1 activity. Other authors report that one possible cause of the PON activity decrease could be the lower HDL and apo-AI levels in CRF patients.[66] Reduced PON1 activity in patients with CRF may indicate antioxidant capacity of HDL. This may increase oxidation of LDL by lipid peroxidation, thereby contributing to the accelerated development of atherosclerosis in CRF.[63] It is also reported that PON1 activity is decreased with increase in severity of renal failure.[63]

Patients on long-term hemodialysis have reduced PON1 activity and this could be related to reduced HDL-cholesterol and apoAI levels.[67] Increased high sensitive C-reactive protein (HS-CRP) associated with abnormal lipoprotein profile, decreased PON1 activity, and increased oxidative stress linked to uremia may contribute to increased cardiovascular risk in people undergoing hemodialysis.[62,68] PON1 activity has shown to correlate well with the oxidative stress markers.[62,68] The PON1 activity is shown to be decreased significantly in hemodialysis patients with or without hepatitis C virus (HCV) infection. Furthermore, presence of HCV infection did not affect the PON1 activity in hemodialysis patients.[69]

PON1 has shown to have thiolactonase activity and physiologically prevent accumulation of homocysteine.[70] Decreased PON1 activity in CRF patients and CRF patients on hemodialysis may increase homocysteine level. This altered mechanism along with reduced renal clearance of homocysteine in CRF patients may cause increased accumulation of homocysteine thiolactone and may augment protein homocysteinylation, which may predispose them to early atherogenesis.[71,72] Although, hemodialysis treatment decreases total homocystein levels by approximately 30-40%, but levels rebound to their elevated pretreatment values.[73] Reports on correlation of homocysteine levels with PON1 activity in CRF patients are not consistent, Janel *et al*.[74] and Greece *et al*.[71] observed an inverse correlation between PON1 activity and homocysteinemia, however no such correlation was observed by Dronca *et al*.[73]

## Conclusion

PON is an enzyme with 354 amino acids and 43 kDa. Of the three isoforms (PON1-3), PON1 has been well studied in different disease conditions; PON1 is an antioxidant and antiatherogenic enzyme situated over HDL. PON1 activity is shown to be decreased in CRF patients, particularly those on hemodialysis, which may increase susceptibility to CVD. Although, the exact cause and effect relation between decrease in PON1 and atherogenesis in CRF patients is not clear, but the reports can lead to development of possible therapeutic target to prevent development of CVD in this patient population.
